# Comparison of the Ability of Artificial-Intelligence-Based Computer-Aided Detection (CAD) Systems and Endoscopists to Detect Colorectal Neoplastic Lesions on Endoscopy Video

**DOI:** 10.3390/jcm12144840

**Published:** 2023-07-22

**Authors:** Yoshitsugu Misumi, Kouichi Nonaka, Miharu Takeuchi, Yu Kamitani, Yasuhiro Uechi, Mai Watanabe, Maiko Kishino, Teppei Omori, Maria Yonezawa, Hajime Isomoto, Katsutoshi Tokushige

**Affiliations:** 1 Department of Digestive Endoscopy, Tokyo Women’s Medical University Hospital, 8-1, Kawada-Chou, Shinjuku-Ku, Tokyo 162-8666, Japan; nonaka513@gmail.com (K.N.); m10058mt@jichi.ac.jp (M.T.); yukamitani@aol.com (Y.K.); uechi.yasuhiro@twmu.ac.jp (Y.U.); watanabe.mai@twmu.ac.jp (M.W.); kishino.ige@twmu.ac.jp (M.K.); 2 Institute of Gastroenterology, Tokyo Women’s Medical University Hospital, 8-1, Kawada-Chou, Shinjuku-Ku, Tokyo 162-8666, Japan; omori.teppei@twmu.ac.jp (T.O.); yonezawa.maria@twmu.ac.jp (M.Y.);; 3 Division of Gastroenterology and Nephrology, Department of Multidisciplinary Internal Medicine, Faculty of Medicine, Tottori University, 36-1, Nishi-Chou, Yonago 683-8504, Japan; isomoto@tottori-u.ac.jp

**Keywords:** computer-aided detection/diagnosis, colorectal polyp, colonoscopy

## Abstract

Artificial-intelligence-based computer-aided diagnosis (CAD) systems have developed remarkably in recent years. These systems can help increase the adenoma detection rate (ADR), an important quality indicator in colonoscopies. While there have been many still-image-based studies on the usefulness of CAD, few have reported on its usefulness using actual clinical videos. However, no studies have compared the CAD group and control groups using the exact same case videos. This study aimed to determine whether CAD or endoscopists were superior in identifying colorectal neoplastic lesions in videos. In this study, we examined 34 lesions from 21 cases. CAD performed better than four of the six endoscopists (three experts and three beginners), including all the beginners. The time to lesion detection with beginners and experts was 2.147 ± 1.118 s and 1.394 ± 0.805 s, respectively, with significant differences between beginners and experts (*p* < 0.001) and between beginners and CAD (both *p* < 0.001). The time to lesion detection was significantly shorter for experts and CAD than for beginners. No significant difference was found between experts and CAD (*p* = 1.000). CAD could be useful as a diagnostic support tool for beginners to bridge the experience gap with experts.

## 1. Introduction

The incidence of colorectal cancer is increasing worldwide [1]. Based on temporal profiles and demographic projections, the number of new cases of colorectal cancer is expected to increase from 1.4 million in 2012 to 2.2 million in 2030 [1]. Many colorectal cancers are known to occur by the adenoma–carcinoma sequence mechanism [2], and colonoscopy resection of adenoma and other neoplastic lesions is known to reduce the mortality rate of colorectal cancer [3,4,5]. Conversely, when neoplastic lesions cannot be detected, the subsequent colorectal cancer risk may increase. The most important cause of post-colonoscopy colorectal cancer (PCCRC), which is a newly identified colorectal cancer after total colonoscopy, is an overlooked neoplastic lesion in previous colonoscopies and accounts for 60–80% of PCCRC causes [6]. Therefore, the adenoma detection rate (ADR), which is defined as the rate of detection of one or more colorectal tumors on screening colonoscopy conducted by a single physician, is an important quality indicator for detecting lesions [7,8]. Kaminski et al. reported that the incidence of PCCRC was significantly lower in patients who underwent colonoscopy by an endoscopist who had a high average ADR of at least 20% [9]; this supports the importance of ADR. Endoscopists have made various efforts to improve their ADR, even slightly, such as conducting good pre-treatment, affixing tip attachments, and taking the appropriate time for observations. In addition to these efforts, the development of image-enhanced endoscopy (IEE) techniques, such as narrow-band imaging (NBI, Olympus Medical Systems, Tokyo, Japan) and blue-laser imaging (BLI, Fujifilm Co., Ltd. Tokyo, Japan), has contributed to increased ADR values [10]. At present, where artificial intelligence has gradually enriched our daily lives, even in the field of endoscopy, computer-aided detection/diagnosis (CAD) systems using artificial intelligence (AI) have been developed in recent years to help further increase ADR [11,12]. The major roles of CAD are computer-aided detection (CADe) and computer-aided diagnosis (CADx). Improved CADe functionality is directly linked to increased ADR, so it has preceded the development of CADx. Many still-image-based studies have been conducted on the usefulness of CAD [13,14]. Sakamoto et al. reported on the validity of CAD using still images extracted from endoscopic videos by experienced endoscopists as validation data [13]. In this report, CADe in white light imaging (WLI) demonstrated a sensitivity of 94.5% and specificity of 87.2%. A study conducted by Weigh et al. using still-image-based analysis reported that non-experts using CADe in combination demonstrated comparable sensitivity but inferior specificity compared to experts [14]. Similar to the still-image-based reports, there have been video-based reports on the usefulness of CADe in real-world clinical practice—but very few. A subsequent study by Wang et al. involved a randomized controlled trial of over 1000 patients, where they reported that the tumor detection rate in the concomitant CADe use group was significantly higher than in the control group [11]. This study is based on video analysis, but the cases included in the CADe group and the control group are completely different. It is expected that when CAD is used in clinical settings, many endoscopists will compare their polyp detection ability in colonoscopies to that of CAD; thus, a scientific assessment of the superiority or inferiority of CAD would be of interest to many endoscopists. In order to scientifically verify this, still-image-based research is not appropriate for actual clinical practice. Even in video-based studies, it is challenging to directly compare the detection capabilities for each tumor lesion within the same case when the evaluation cases differ between the comparison groups. In this study, we simultaneously recorded endoscopy videos with and without concomitant CAD, which enabled the comparison of the ability of an endoscopist and CAD to detect colorectal neoplastic lesions in the exact same case. This study aimed to compare the abilities of CAD and endoscopists to detect exactly the same colorectal neoplastic lesions on endoscopy video.

## 2. Materials and Methods

### 2.1. Study Design

This video-based validation study involved a single center in Japan—Tokyo Women’s Medical University Hospital. Endoscopy videos were used to evaluate whether CAD EYE (FUJIFILM Medical, Tokyo, Japan), an AI-equipped colonoscope CAD system, was superior to endoscopists in its ability to detect colorectal neoplastic lesions, and to compare the times needed by CAD EYE and the endoscopists to detect tumors. This study was approved by the ethics committee of Tokyo Women’s Medical University. Informed consent was obtained from all patients for using the videos. Patients were free to withdraw consent at any time. In addition to the study coordinator, Endoscopist A (Y.M.), a total of six people participated in this study—three experts (>5000 colonoscopies) and three beginners (<500 colonoscopies). Personal information on the six participating endoscopists will not be disclosed. Similar to what was carried out with the patients, we obtained informed consent from the six endoscopists who participated, with the freedom to withdraw consent at any time. The study was registered in the UMIN clinical trial registry (UMIN000056123).

### 2.2. CAD EYE (CADe and CADx)

**Figure 1:**
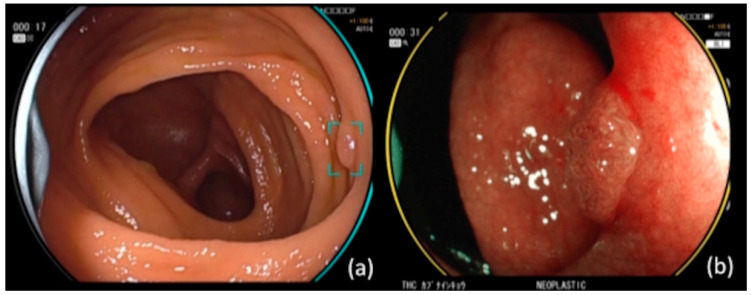
(**a**) CAD EYE endoscope with CADe function. The device detects lesions and displays targets. In this case, the target remained visible because there was a lesion. (**b**) CAD EYE endoscope with CADx function. Switching to BLI shows a qualitative diagnosis of the lesion. Since the lesion was neoplastic, it displayed a yellow circular flame.

CAD EYE is a previously mentioned type of commercialized CAD system that was developed using deep learning, and it is the first CAD system to have both CADe and CADx on the same platform. It is trained with accurate and high-quality information and a large number of images, and it has been reported to be a CAD that achieves high sensitivity comparable to that of an endoscopist and has few false positives [15]. Additionally, it achieves high-speed processing of 60 images per second (60 fps). It was first sold in Europe, with sales commencing in Japan in November 2020. The installation of AI software in the function expansion unit EX-1 (Fujifilm, Tokyo, Japan), which was sold at the same time, enabled its use with the 700, L600, and 6000 system scopes, which are the company’s colonoscopes. This can also be used not only with white light imaging (WLI) but also with linked-color imaging (LCI, Fujifilm Co., Ltd., Tokyo, Japan). In addition, it is possible to switch between CAD functions at the touch of a button in real-time without a freeze operation (Figure 1). The CAD EYE colonoscope equipped with AI used in this study uses CADe for detecting lesions and CADx for performing qualitative diagnoses. The CADe function can detect non-neoplastic lesions such as hyperplastic polyps. A target is displayed on lesions with low confidence levels, and a warning tone sounds at high confidence levels. If there is indeed a lesion, the target often remains visible. On the other hand, in the case of false positives, the target often disappears quickly. The CADx function switches to blue laser/light imaging (BLI) in the unenlarged state to differentiate between neoplastic and hyperplastic lesions. The screen does not need to be frozen or magnified, and it does not interfere with observations since it operates in real time. It displays a yellow circular frame for neoplastic lesions and a green circle for hyperplastic lesions, making it easy to visually grasp the results. The EX-1 is equipped with a video recording function, but it is usually impossible to simultaneously record videos that concomitantly use and do not use CAD, and special innovations are needed for simultaneous recording.

The effectiveness of the CAD EYE program has been evaluated by conducting a retrospective performance test using static endoscopy images obtained at four centers in Europe and three centers in Japan for colorectal polyps [13,14]. CADe had detection sensitivity values of 94.5% and 96% for WLI and LCI, respectively. CADx can be used in both the WLI and BLI modes, and their accuracy rates are high at 93.2% and 94.9%, respectively, which demonstrates the usefulness of this system.

### 2.3. Study Population

**Figure 2:**
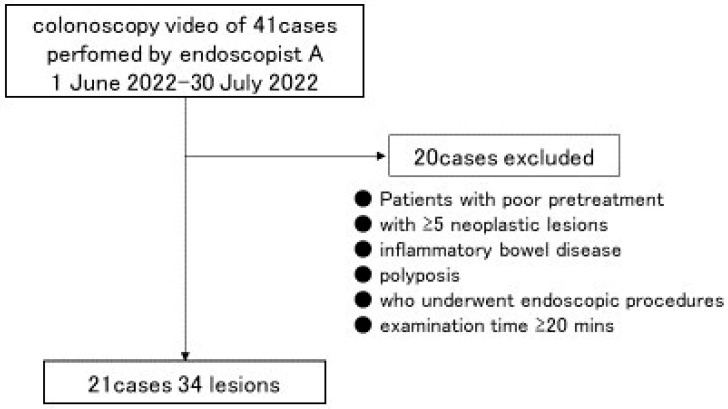
Study population.

The study used all 41 colonoscopies performed by expert Endoscopist A (Y.M.), who is a Board Certified Fellow and Trainer of the Japan Gastroenterological Endoscopy Society at Tokyo Women’s Medical University from 1 June to 30 July 2022, using a high-definition colonoscope (EC-760ZP-V/M, FUJIFILM Medical, Tokyo, Japan) and a high-definition monitor (CuratOR EX2621, EIZO Corporation, Hakusan, Japan). To avoid selection bias, we did not examine the symptoms, purpose of being examined, age, or sex of any of the patients. Referring to the previous report [16], the inclusion criteria were the presence of at least one neoplastic lesion (adenoma or adenocarcinoma) measuring < 10 mm. For the final determination of whether the lesion was neoplastic, the gold standard was CAD EYE’s CADx diagnosis. Patients with poor pretreatment (0 or 1 in any segment on the Boston Bowel Preparation Scale), with ≥5 neoplastic lesions, with inflammatory bowel disease, with polyposis, who underwent endoscopic procedures that included biopsies, and whose examination took ≥20 min were excluded. Finally, 21 patients with 34 lesions were included (Figure 2).

### 2.4. Preparation of the Colonoscopy Videos

In all 41 colonoscopies, two video types were recorded simultaneously: one with the CADe function of CAD EYE and one without CADe. WLI was used for observation instead of the concomitant use of IEE in all cases when searching for lesions. All patient data, such as the date and ID, was removed from the videos. Unlike actual clinical practice, the alarm sound was not recorded in the video with CADe. When the CADe function detected a lesion, the CADx function was used in combination with CADe to make a qualitative diagnosis of whether it was neoplastic.

### 2.5. Time Measurement and Comparison

The lesion detection time of CAD was measured and recorded when Endoscopist A (Y.M.) viewed the video with CADe, from when the cecum was reached to when the lesion was detected, and the target was displayed. The six other endoscopists viewed the videos without CADe, and their lap times from the cecum to recognizing a lesion as neoplastic were recorded. The six endoscopists were informed in advance that the videos contained 1–4 neoplastic lesions. To ensure uniformity of the time measurements, they began recording a still image 5 s after reaching the cecum, and when the still image was released, the timer of the light source also began recording. The timer was recorded in both the videos with and without CADe. The start button on the stopwatch was pressed at the moment the timer displayed “0:00” to begin measuring time. The endoscopists were allowed to identify more than 4 lesions during the procedure, and even if over this amount was detected, the endoscopists had to continue viewing the scope until it was removed from the anus. The time required to identify each lesion was compared between CAD and each endoscopist. With the time required by CAD as the reference, (time required by the endoscopist)—(time required by CAD) = time X was recorded for each polyp, with the CAD winning if time X > 0.3 s and the endoscopist winning if time X ≤ 0.3 s. If the endoscopists could not identify a lesion, they were deemed to have lost. Each video was viewed only once from start to finish without interruption, and the times were recorded. The number of cases viewed per day was limited to 3, and no special restrictions have been placed on the date and time of viewing videos. A high-performance stopwatch (Seiko Stopwatch, SOLER STANDARD SVAJ001) capable of measuring to the 1/100th of a second was used to measure time, and the endoscopists used their own laptop monitors.

#### 2.5.1. Basis for Setting Time X of Win/Loss Decision

When performing colonoscopy with CAD in actual practice, endoscopists feel that they can detect a lesion faster than CAD when the moment they recognize the lesion comes before the CAD alert sound. In the present study, due to the difficulty of reproducing the alert sound, we compared the moment when the endoscopist recognized the lesion with the time when the CAD system displayed the target. It is reported that the simple reaction time of a human is about 0.2 s [17]. Therefore, we considered the CAD confidence assessment process/sound processing time and the reaction time for the endoscopist to press the stopwatch, resulting in 0.3 s.

#### 2.5.2. False Positives

CAD false positives were defined as the number of times CAD displayed a target at a site where no lesion was present with reproducibility, and the endoscopist false positives were defined as the number of times the endoscopist pressed the stopwatch at a site where no lesion was present. The number of false positives for CAD and each endoscopist was recorded, where false positive A was defined as when non-neoplastic lesions, such as hyperplastic polyps or lipoma, were present, whereas false positive B was defined as when no lesions were present. The false positive A and B assessments for CAD were performed by the coordinator (Endoscopist A) by viewing the videos with CADe. Assessments of false positive A and B for the endoscopists were made by Endoscopist A by viewing the videos without CADe and referencing the lap times recorded by each endoscopist.

### 2.6. Polyp Characteristics

The size, localization, and macroscopic type of the target lesions were recorded. Polyps that were “easily detected by endoscopists” were defined as those in which at least four of the six endoscopists beat CAD, and those that “tended to be missed by endoscopists” were those that were missed by at least one endoscopist.

### 2.7. Outcome Measures

The primary endpoints were CAD and endoscopist wins (number of wins for each endoscopist). Secondary endpoints were the difference in time required for CAD and beginners, the difference in time required for CAD and experts, the difference in time required for beginners and experts, the difference in the number of false positives between CAD and endoscopists, characteristics of polyps that were easily detected by endoscopists, and characteristics of those that tended to be missed by endoscopists.

### 2.8. Statistical Analysis

The primary endpoint was analyzed using a histogram. A one-way repeated measures analysis of variance was used to compare the differences in time to lesion detection between the CAD, beginner, and expert groups. The Bonferroni method was used for multiple comparisons. An unpaired *t*-test was used to compare beginner-CAD and expert-CAD for the total number of false positives and the number of false positives A and B in the CAD, beginner, and expert groups. The Bonferroni method was used to correct the significance level to avoid multiplicity. To examine the characteristics of polyps that were easily detected by endoscopists, we classified the data into two groups—polyps that were easily detected by endoscopists and other data—and then compared their size, location, and shape. A similar comparison was made for polyps endoscopists tended to miss, with a chi-square test used to compare groups. Adjusted residuals were used for comparisons between groups. SPSS ver. 29.0 was used for all statistical analyses except histograms. Statistical significance was set at 5%.

## 3. Results

### 3.1. Lesion Characteristic

The lesion size was 1–5 mm in 82% (28/34) and 6–10 mm in 18% (6/34). The location was 2.9% (1/34) in the cecum, 29.4% (10/34) in the ascending colon, 35.3% (12/34) in the transverse colon, 17.6% (6/34) in the descending colon, 8.8% (3/34) in the sigmoid colon, and 5.9% (2/34) in the rectum. The macroscopic type was the slightly elevated type (Paris type IIa) in 73.5% (25/34) and the protruded type (Paris type Is) in 26.4% (9/34) (Table 1).

#### Polyps That are Easy for Endoscopists to Detect or Those They Tend to Miss

**Figure 3:**
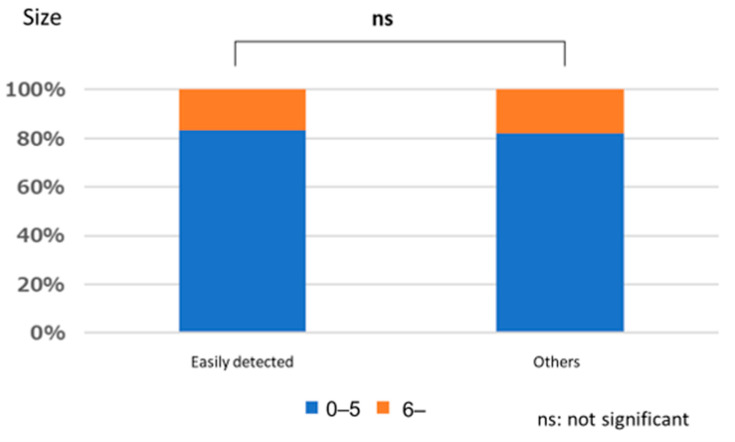
Size characteristics of easily detected polyp.

**Figure 4:**
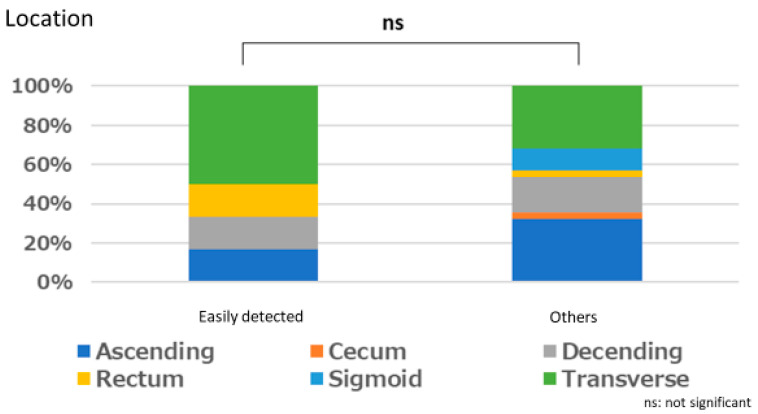
Location characteristics of easily detected polyp.

**Figure 5:**
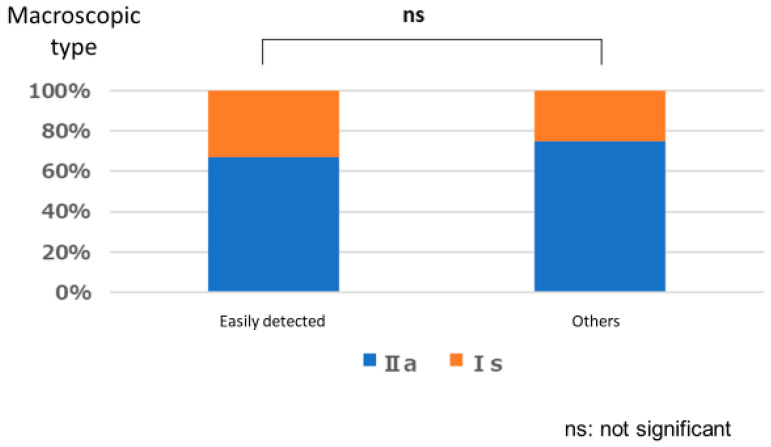
Macroscopic type characteristics of easily detected polyp.

**Figure 6:**
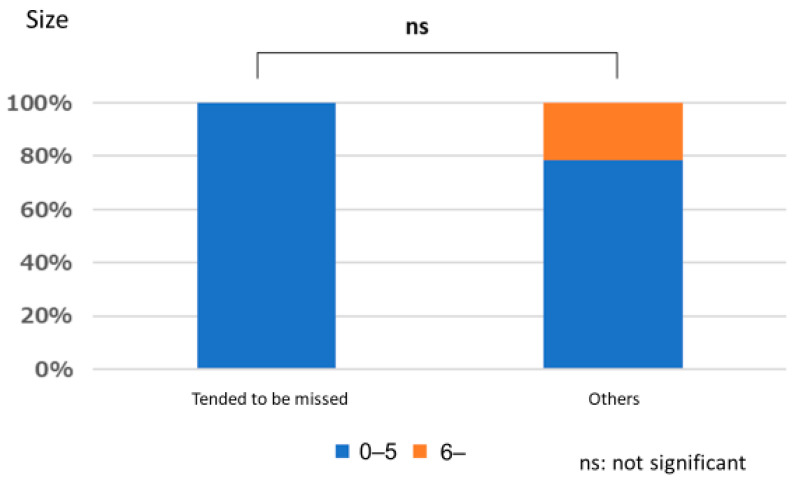
Size characteristics of polyp that tended to be missed.

**Figure 7:**
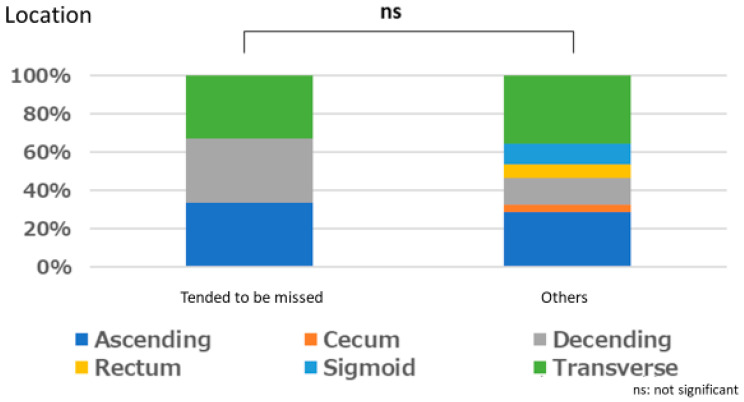
Location characteristics of polyps that tended to be missed.

**Figure 8:**
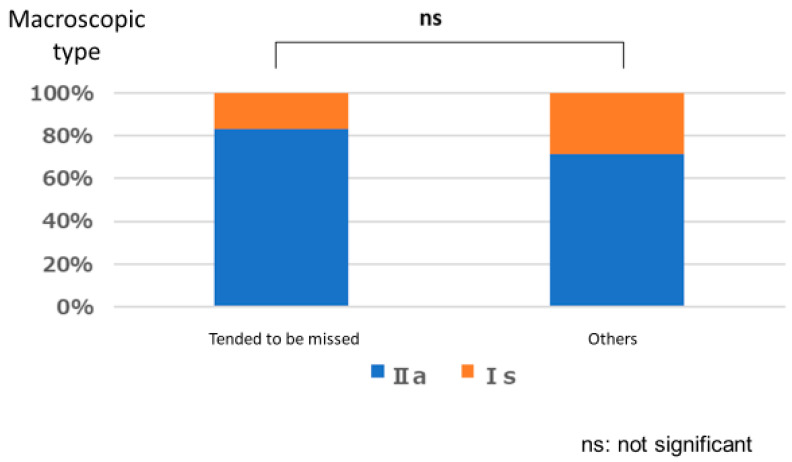
Macroscopic type characteristics of polyps that tended to be missed.

There was no significant relationship between polyps that are easily detected by endoscopists and lesion size (*p* = 1.000), location (*p* = 0.667), or macroscopic type (*p* = 1.000). Similarly, no significant relationships were observed between polyps that tended to be missed and size (*p* = 0.333), location (*p* = 0.661), and macroscopic type (*p* = 0.661) (Figure 3, Figure 4, Figure 5, Figure 6, Figure 7 and Figure 8).

### 3.2. Lesion Detection Time

#### 3.2.1. Number of Endoscopist Wins

**Figure 9:**
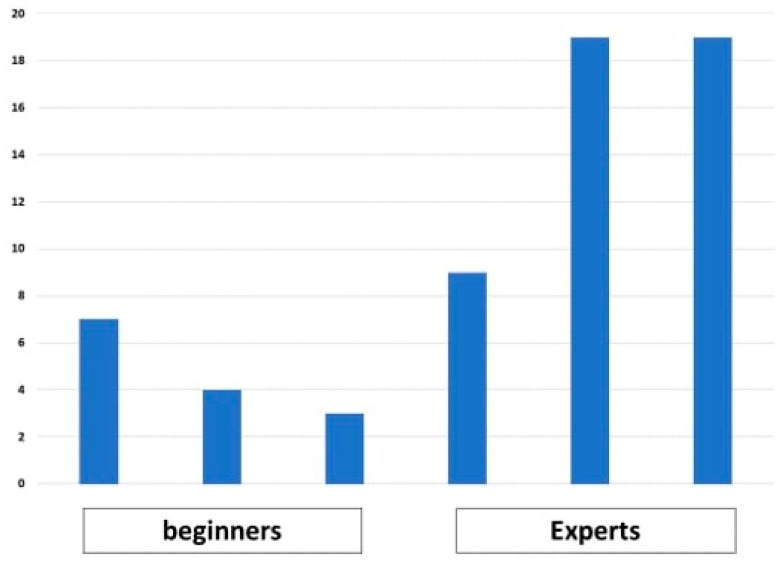
Number of wins over CAD for each endoscopist (34 lesions).

The time it took three beginners and three experts to identify 34 lesions was measured. As shown in Figure 9, the beginners had three, four, and seven wins over CAD. Meanwhile, the experts had nine, nineteen, and nineteen wins over CAD.

#### 3.2.2. Group Comparisons of Time Required to Detect Lesions (Time X)

**Figure 10:**
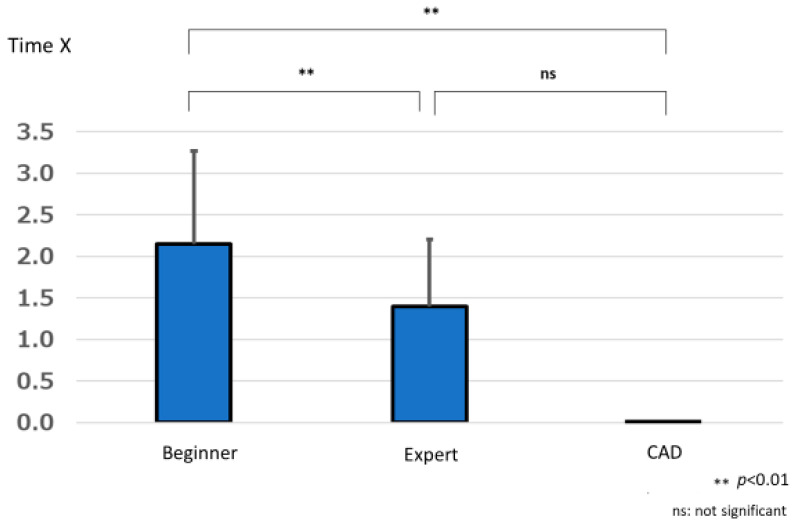
Difference in time required for CAD and endoscopists to detect lesions (Time X).

The difference in the time to lesion detection by beginners was 2.147 ± 1.118 s, that by experts was 1.394 ± 0.805 s, and that by CAD was 0.000 ± 0.000 s. One-way repeated measures analysis of variance showed a significant difference in the lesion detection times between the beginner, expert, and CAD groups (F (1.612, 166) = 18.787, *p* < 0.001). Multiple comparisons using the Bonferroni method showed significant differences between beginners and experts (*p* < 0.001) and beginners and CAD (*p* < 0.001), with experts and CAD having significantly shorter times for lesion detection compared to beginners. No significant difference was found between experts and CAD (*p* = 1.000) (Figure 10).

### 3.3. Number of False Positives

#### 3.3.1. Comparison of the Number of False Positives by Group

**Figure 11:**
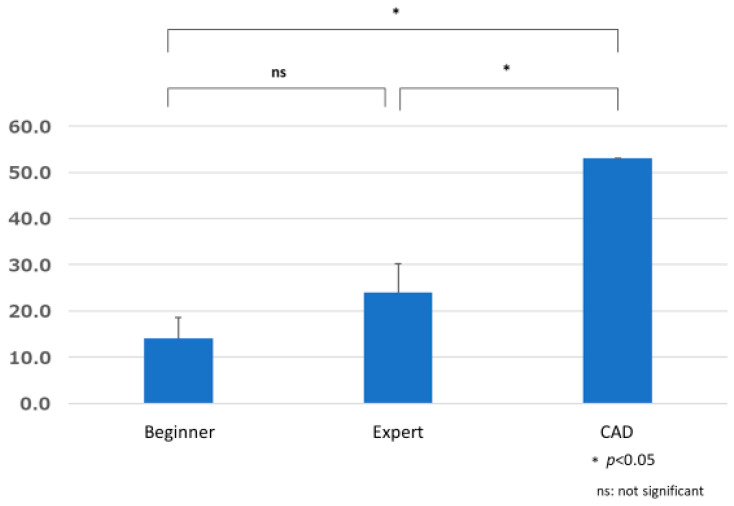
Comparison of total number of false positives.

The total number of false positives 14.000 ± 4.583 for the beginner group, 24.000 ± 6.245 for the expert group, and 53 for CAD. The results of one-sample and unpaired *t*-tests showed significant differences between the beginner group and CAD (*p* = 0.014) and between the expert group and CAD (*p* = 0.045), with significantly more false positives for CAD than for the beginner and expert groups. There was no significant difference between the beginner and expert groups (*p* = 0.267) (Figure 11).

#### 3.3.2. Comparison of False Positive A by Group

**Figure 12:**
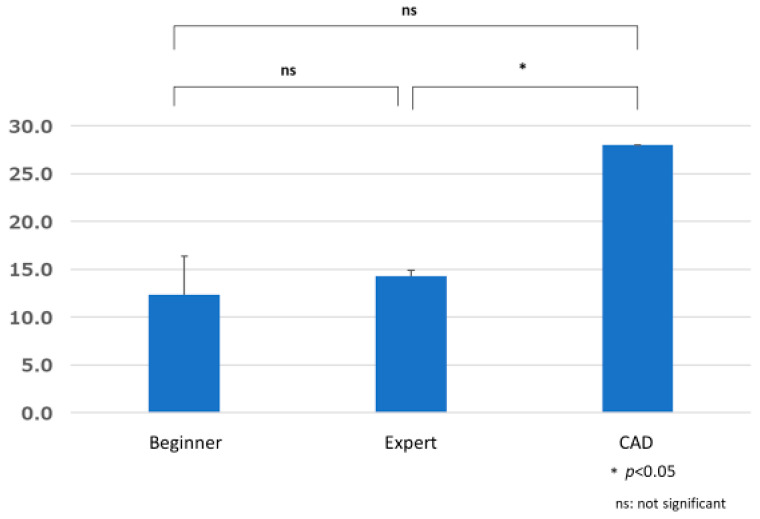
Comparison of false positive A by groups.

The number of false positive A was 12.333 ± 4.041 in the beginner group, 14.333 ± 0.577 in the expert group, and 28 in the CAD group. The results of one-sample and unpaired *t*-tests showed a significant difference only between the expert and CAD groups (*p* = 0.002), with the latter having significantly more false positive A. There were no significant differences between the beginner and expert groups (*p* = 1.000) or the beginner and CAD groups (*p* = 0.064) (Figure 12). The 28 false positive A by CAD included 25 hyperplastic polyps, 2 lipomas, and 1 angioectasia (Table 2).

#### 3.3.3. Comparison of False Positive B by Group

**Figure 13:**
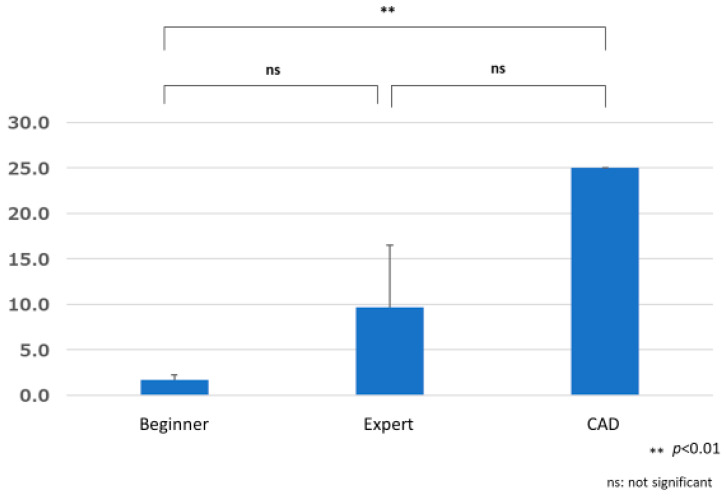
Comparison of false positive B by group.

The number of false positive B in the beginner group was 1.667 ± 0.577, 9.667 ± 6.807 in the expert group, and 25 in the CAD group. The results of one-sample and unpaired *t*-tests showed a significant difference only between the beginner and CAD groups (*p* = 0.001), with the latter having significantly more false positive B. There were no significant differences between the beginner and expert groups (*p* = 0.534) or between the expert and CAD groups (*p* = 0.180) (Figure 13).

## 4. Discussion

In this study, CAD won for more than half of the 34 lesions against four of the six endoscopists. In particular, CAD won against all three beginners. In addition, while there was no significant difference in the time needed for lesion detection between the CAD and expert groups, there was a significant difference of more than 2 s between the CAD and beginner groups, with the beginners being slower. Koh et al. reported that even experienced endoscopists can improve on baseline ADR when using CAD [18]. Xu et al. similarly observed an improvement in ADR with CAD in both expert and non-expert patients [19]. Both reports differ in that they compare ADR in the combined CAD and no CAD groups, whereas the present study compares CAD itself and the endoscopist’s ability to detect colorectal neoplastic lesions. In the present study, the ability of CAD EYE to detect lesions was not superior to that of experts, but it was superior to beginners. There have been reports that beginners have improved their ability to detect neoplastic lesions using CAD functions, gaining lesion detection abilities equivalent to experts [14,20]. Hence, it is also possible that the time required for beginners to detect lesions could be reduced using CAD.

In contrast, the number of false positives was higher with CAD than with the experts or beginners. There is known to be a trade-off between speed and accuracy in reaction [21], which was especially evident between beginners and CAD. In addition, most of the false positive A by CAD were hyperplastic polyps. The CADe function of CAD EYE was originally designed to pick up hyperplastic polyps as well, and because the present study defined hyperplastic polyps as false positives, it is inevitable that CAD would have more false positive A than the experts. While there are concerns that using the CADe function may lead to more false positives [22], in actual clinical practice, it is often impossible to differentiate adenoma and hyperplastic polyps on initial presentation, and they can only be distinguished in close examinations. Therefore, casting a wide net and picking up hyperplastic polyps is an important support tool for not overlooking neoplastic lesions. Moreover, there were significantly more instances of false positive B with CAD than the beginners. Most false positive B instances by CAD were artifacts due to mucosal flexures, which is similar to previously reported results [22].

There have been various studies on the ease of detecting different types of lesions, including reports that CADe is inferior in detecting small flat lesions, but also that it is better at detecting small adenomas [11,23]. In addition, a meta-analysis of randomized clinical trials by Huang et al. reported that CADe was advantageous in pointing out lesions except for lesions localized in the cecum and stromal lesions [24]. However, in the present study, there was no particular trend with regard to lesions that were easy for endoscopists to detect and those they tended to miss, suggesting the possibility that factors related to the endoscopist influence lesion detection more than factors related to the lesion.

This study has several limitations. First, it was a small, single-center study. Second, in a few cases, Endoscopist A (Y.M.), who performed all the colonoscopies, failed to recognize a neoplastic lesion despite the presence of one. The presence of these may affect the mean tumor detection time and the number of false positives. Third, although the research target lesions were neoplastic lesions, the gold standard for qualitative diagnosis of lesions was endoscopic diagnosis using CADx for lesions that have not been pathologically proven to be neoplastic. Fourth, a competitive spirit on the part of the endoscopists, who do not want to be slower than CAD, may have impacted the lesion detection time and false positive detection time, creating a discrepancy with what would occur in actual clinical practice. Fifth, the measurement environment for the endoscopists was not uniform. For the sake of concentration capacity, we limited the number of videos viewed to three per day, although we did not specify the time of day when the videos were viewed. The fatigue level may affect the reaction time, and the fatigue levels of each endoscopist may have had an impact on the results. Because the same monitor was not used, differences in resolution may have impacted video playback due to the limitations of the facility’s environment. Finally, the study was conducted using a FUJIFILM endoscope equipped with CAD. Future studies should be conducted using endoscopic systems from different companies and using image enhancement endoscopy such as blue laser imaging and LCI in combination with conventional light observations.

## 5. Conclusions

In this study, the ability of CAD EYE to detect colorectal neoplastic lesions was comparable to that of an expert but superior to that of a beginner. The lesion detection capability achieved through deep learning in computer-aided diagnosis (CAD) can be said to be comparable to the expertise developed by experienced experts over many years. For beginners, CAD can serve as a tool that can help bridge the experience gap with experts. This study compared the ability of CAD and endoscopists to detect colorectal neoplastic lesions under WLI observation. As mentioned in the discussion, similar studies are expected in the future under the combined use of IEE techniques such as BLI, LCI, and NBI.

## Figures and Tables

**Table 1:** Lesion characteristics.

**Size**	
0–5 mm	8
6–10 mm	26
**Location**	
Cecum	1
Ascending colon	10
Transverse colon	12
Descending colon	6
Sigmoid colon	3
Rectum	2
**Shape**	
Is	9
IIa	25

**Table 2:** Breakdown of false positive A.

Hyperplastic Polyp	25
Lipoma	2
Angioectasia	1

## Data Availability

The data underlying this article will be shared upon reasonable request to the first or corresponding authors.
